# Calculating Filament Feed in the Fused Deposition Modeling Process to Correctly Print Continuous Fiber Composites in Curved Paths

**DOI:** 10.3390/ma13204480

**Published:** 2020-10-09

**Authors:** Behnam Akhoundi, Mojtaba Nabipour, Faramarz Hajami, Shahab S. Band, Amir Mosavi

**Affiliations:** 1 Additive Manufacturing Laboratory, Faculty of Mechanical Engineering, Tarbiat Modares University, Tehran 14115-143, Iran; behnam.akhoundi@modares.ac.ir (B.A.); mojtaba.nabipour@modares.ac.ir (M.N.); 2 Department of Mechanical Engineering, Faculty of Mechatronics, Karaj Branch, Islamic Azad University, Karaj 3149968111, Iran; faramarz.hajami@kiau.ac.ir; 3 Institute of Research and Development, Duy Tan University, Da Nang 550000, Vietnam; 4 Future Technology Research Center, College of Future, National Yunlin University of Science and Technology 123 University Road, Section 3, Douliou, Yunlin 64002, Taiwan; 5 Faculty of Civil Engineering, Technische Universität Dresden, 01069 Dresden, Germany; 6 School of Economics and Business, Norwegian University of Life Sciences, 1430 Ås, Norway; 7 Kando Kalman Faculty of Electrical Engineering, Obuda University, 1034 Budapest, Hungary

**Keywords:** fused deposition modeling (FDM), 3D printing, filament feed, curvilinear path, variable-stiffness composites, computational mechanics, Composite material, material design, additive manufacturing (AM), computational materials science

## Abstract

Fused deposition modeling (FDM) is a popular additive manufacturing (AM) method that has attracted the attention of various industries due to its simplicity, cheapness, ability to produce complex geometric shapes, and high production speed. One of the effective parameters in this process is the filament feed presented in the production G-code. The filament feed is calculated according to the layer height, the extrusion width, and the length of the printing path. All required motion paths and filling patterns created by commercial software are a set of straight lines or circular arcs placed next to each other at a fixed distance. In special curved paths, the distance of adjacent paths is not equal at different points, and due to the weakness of common commercial software, it is not possible to create curved paths for proper printing. The creation of a special computer code that can be used to make various functions of curved paths was investigated in this study. The filament feed parameter was also studied in detail. Next, by introducing a correction technique, the filament feed was changed on the curved path to uniformly distribute the polymer material. Variable-stiffness composite samples consisting of curved fibers can be produced with the proposed method. The high quality of the printed samples confirms the suggested code and technique.

## 1. Introduction

In recent years, additive manufacturing (AM) techniques have made it possible to produce complex parts with the desired geometric shape without needing to use special tools [1]. Unlike subtractive manufacturing and machining methods, AM forms parts layer by layer [2]. Fused deposition modeling (FDM), a special kind of AM technique, has been popular among personal and industrial users because of the simplicity of the process, the high reliability, and the ability to produce complex parts from thermoplastic materials [3,4,5,6]. Despite significant advantages, the process has some limitations, and extensive studies were conducted to overcome these problems [1,7,8,9]. The main limitations of this method are directional mechanical properties, material restrictions, low dimensional accuracy and surface finish, lower strength compared to other production methods, and weakness in commercial software [10,11,12]. Most studies were designed to improve the mechanical properties and optimize the parameters of this process [13,14]. Some groups studied different materials [1,12,15] and feeding mechanisms with the melting and extrusion consideration system [16], surface finish and dimensional accuracy [17], material swelling [18], in-process cooling [19], thermal analysis of layers [2], and melt stability [20]. Other groups examined the effect of printing parameters such as nozzle temperature, bed temperature, nozzle diameter, nozzle geometry, layer height, extrusion width, sample orientation, raster angle, filling percentage, filling pattern, air gap, and printing speed [3,21]. With considerable improvements in FDM, recent and novel studies emerged, such as producing composites with continuous fibers (which have significant effects on mechanical properties) [11,22,23] or optimizing the nozzle path and filling pattern (which have influential impacts on mechanical properties, dimensional accuracy, and surface smoothness) [24,25].

Baich et al. investigated the effects of low, high, double dense, and solid filling patterns on tensile, flexural, and compressive strengths and moduli [26]. Jin et al. employed the parallel-based generation method to create optimal paths in the process [27]. Akhoundi and Behravesh conducted thermal analysis on filling patterns (concentric, rectilinear, honeycomb, and Hilbert curve) and examined their effects on the tensile and flexural properties of printed samples [2]. Koch et al. investigated the mechanical anisotropy of printed products by customized tool path generation [28]. Guan et al. studied the effect of a fill gap on the flexural strength of parts fabricated by curved layer fused deposition modeling [29]. Kumar et al. examined the mechanical properties of printed samples by creating paths based on fractal curves [30].

The instructions for printing a sample and determining the motion path of a nozzle in FDM are given as a single code to the 3D printer. All necessary settings such as nozzle and bed temperature, all movement paths, the filling pattern and percentage, fan speed, printing speed, filament feed, etc. are provided by commercial software in the form of standard codes. One of the critical parameters in G-code is the filament feed, which is calculated according to the extrusion width, the layer height, and the length of the printing path. The filament feed (based on these three parameters) is always constant and it is not possible to change it in different sections of the program. Available commercial software implements various filling patterns such as linear, rectilinear, grid, triangular, star, cube, concentric, honeycomb, 3D honeycomb, Hilbert curve, Archimedes curve, spiral, etc. All of the paths in these patterns provided by commercial software are a set of straight lines or circular arcs that are placed next to each other at a uniform distance. In some cases, such as the production of composites with curved fibers, which are variable-stiffness composites, it is necessary to produce curved paths for the nozzle motion. Due to the weakness of current commercial software (Makerbot, Repetier, Simplify3D, Creatbot, Cura, Slic3r, etc.), it is not possible to create curved paths for proper printing. Since the distance between adjacent paths is not equal at different points when printing curved ones, it is vital to change the amount of filament feed along the nozzle motion so the polymeric materials are uniformly distributed throughout the curved path for proper printing. If there are continuous fibers in the nozzle outlet, the filament feed must also be reduced by a certain amount according to the diameter of the fibers [11].

Due to the aforementioned limitation of the inability of commercial software to create curved paths as well as the constant value of the filament feed or extrusion width throughout the entire produced G-code program, the main goal of this study was to construct a special computer code through which various functions of curved paths can be created. Since the filament feed is variable along curved paths (depending on the path type), achieving the uniform distribution of polymeric materials was another purpose of the study. With uniform distribution of polymeric materials, the continuous fibers can be deposited in curved paths to produce composites with curved fibers.

## 2. Materials and Methods 

In this study, polylactic acid with a diameter of 1.75 mm made by DigitMakers (Toronto, ON, Canada) and E-glass fibers were used to produce the composites. The tex of the main yarn was 800 and the diameter of each glass fiber was 8 µm. The diameter of the glass yarn was about 225 µm [10]. A quantum FDM 3D printer type 2020 (Tehran, Iran) with a bed size of 195 × 195 × 200 mm was used to print the products. The nozzle and bed temperatures were set to 210 and 50 °C, respectively, and MATLAB software was employed to create the necessary program for printing composites.

### 2.1 Generating G-Code

In the first step to generate the G-code required for the FDM 3D printer, path points must be identified. Since the goal is to create curved paths, the path function and its points must be identified and then the filament feed calculated according to the specified points and path lengths. The distance between adjacent paths may vary at different points, so the filament feed must be determined according to each specific path before the final G-code can be generated. Consideration should also be given to the flow percentage in the program to properly print composites with continuous fibers.

#### 2.1.1. Creating Curved Paths

**Figure 1:**
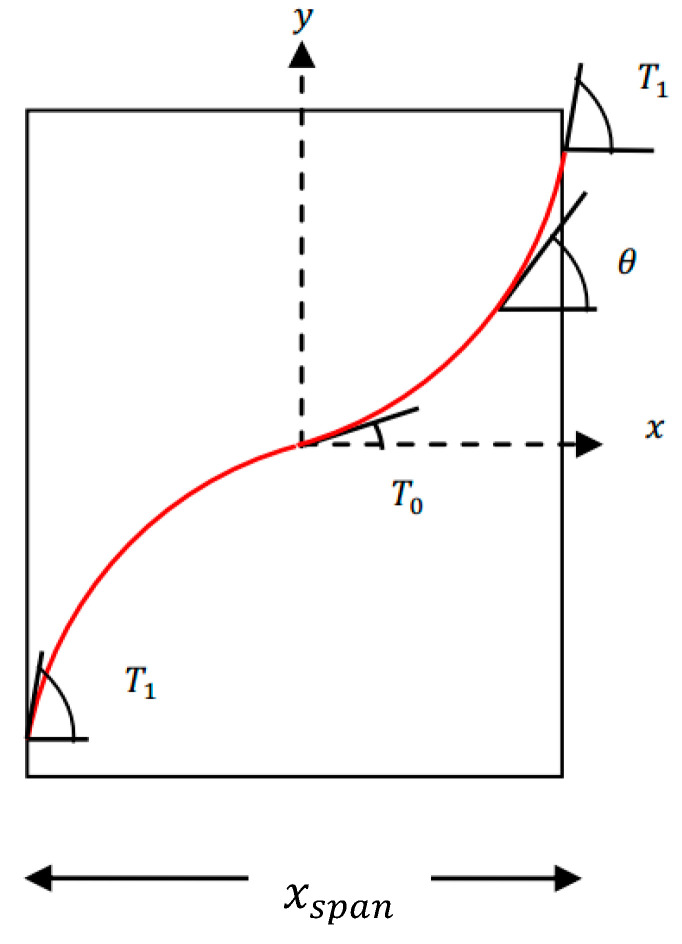
Reference curve with determining parameters. θ is the angle of the fibers in the transfer mode on the *y* axis, *T*_0_ is the center angle of the reference curve, *T*_1_ is the angle of the side edges, *x* is the coordinate of each point along the *x* axis, and *x_span_* is the range of *x*.

The reference curve in Figure 1, which is adapted from [31], was used to produce composites consisting of curved fibers via the transfer method with an automated fiber placement machine. The path equation of the curve is presented in terms of angles at the side edges and at the center. The angle of the curve at the side edges is $T_{1}$ and at the center is $T_{0}$_._ If the path is moved along the y axis, the path angle changes along the *x* axis and remains constant on the y axis. Therefore, by defining the relation of the changing angle of the reference curve in terms of x, the angle of the fibers at any point on the plane can be easily calculated.

According to [31], the Equation (1) can be used to change the angle of the fibers in the transfer mode linearly on the *y* axis:

(1) $$\theta \left(x\right)=T_{0}+2\left(T_{1}-T_{0}\right)\left(\left|x\right|/x_{span}\right)$$

where $T_{0}$ is the center angle of the reference curve, $T_{1}$ is the angle of the side edges, *x* is the coordinate of each point along the horizontal axis, and $x_{span}$ is the range of x. Points from the curved path are needed to generate G-code. If the curved path is a curvilinear function, the slope of the line can only be determined for certain coordinates. Therefore the differential equation in Equation (2) needs be solved to extract the coordinates of the path.

(2) $$dy/dx=\tan^{-1}(T_{0}+2\left(T_{1}-T_{0}\right)\left(\left|x\right|/x_{span}\right))$$

This equation was solved using the Runge–Kutta method for each iteration to obtain the coordinates of the path points. The filament feed needed to be calculated by determining the points from the curved path.

#### 2.1.2. Calculating Filament Feed

**Figure 2:**
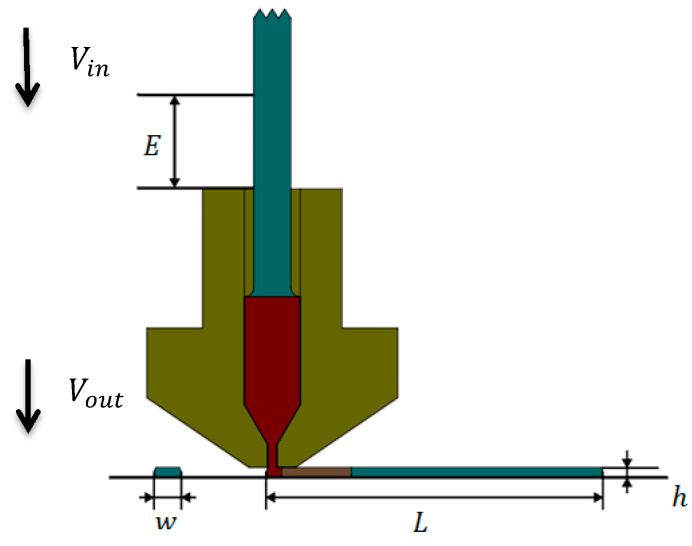
The deposited raster with the specified extrusion width (w) and layer height (h ) in the specified length (L ). $V_{in}$ is the volume of material entering the inlet and $V_{out}$ is the volume of material leaving the outlet.

The volume of material entering the inlet ($V_{in}$) was made equal to the volume of material leaving the outlet ($V_{out}$) by assuming the density changes with temperature were negligible (Figure 2). The value of the filament feed was obtained with Equation (3) by determining the parameters of layer height (h), extrusion width (w), length of the deposited raster (L), and filament diameter (D).

(3) $$E=\left(4w\cdot h\cdot L\right)/\pi D^{2}$$

**Figure 3:**
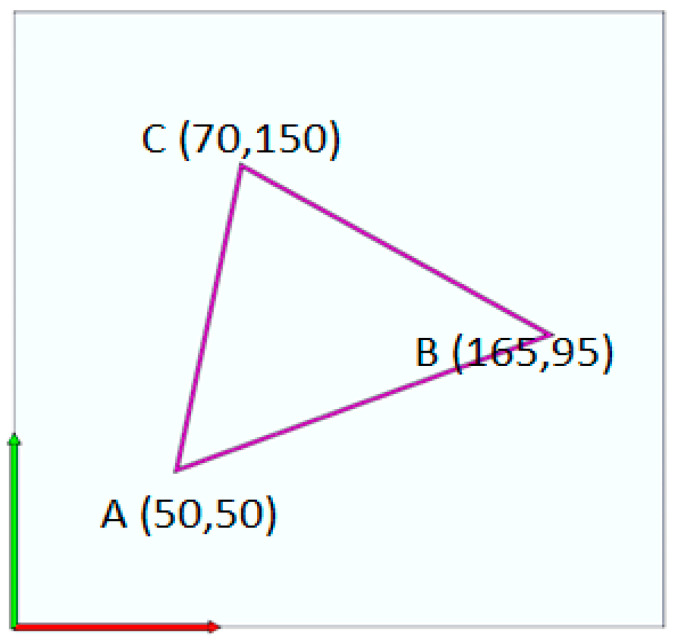
Triangular path created to calculate the filament feed.

The path in Figure 3 was created for printing, where the nozzle moved from point A to B, then from point B to C, and finally from point C to A. The results in Table 1 were obtained by assuming an extrusion width of 0.5 mm and a layer height of 0.2 mm.

The program for this path was written based on the filament feed in both continuous and discrete forms. In the continuous program, the value of E was cumulative, with each line of the program added to the previous value. The continuous program was computed with Equation (4).

(4) *G*0→*X*50→*Y*50 *G*1→*X*165→*Y*95→*E*5.1342 *G*1→*X*70→*Y*150→*E*9.6980 *G*1→*X*50→*Y*50→*E*13.9379

The discrete program was based on Equation (5), where G0 shows the nozzle movement without depositing, *G1* indicates the nozzle movement by depositing, X and Y are the coordinates of each point, *E* is the filament feed, and G92 resets the filament feed for the next line in the program.

(5) *G*0→*X*50→*Y*50 *G*1→*X*165→*Y*95→*E*5.1342 *G*92→*E*0 *G*1→*X*70→*Y*150→*E*4.5638 *G*92→*E*0 *G*1→*X*50→*Y*50→*E*4.2399

#### 2.1.3. Calculating Filament Feed for Curved Paths

**Figure 4:**
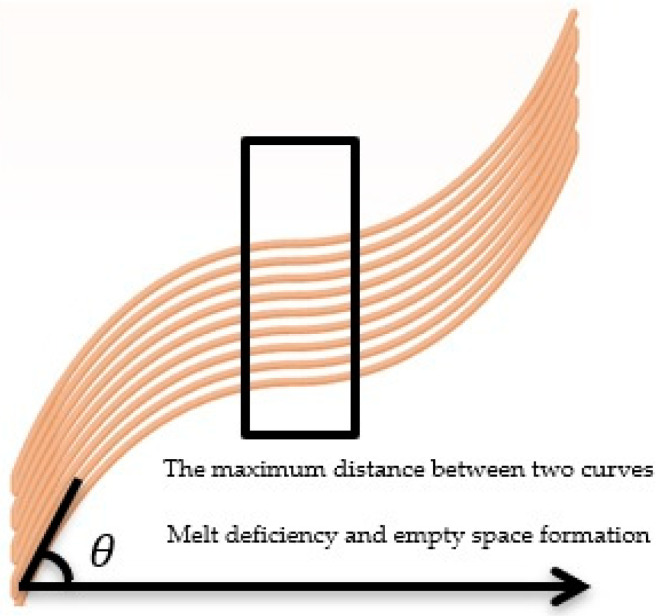
Curved paths with a start and end angle of 70° and a center angle of 0°.

When a curved function was transferred in a certain direction, the vertical distances between two adjacent curved paths was no longer the same. As the fibers angled closer to 90° in the special curvilinear function, the vertical distance between the two paths decreased. At 0°, the vertical distance was equal to the transfer value. Figure 4 presents a curved path with a start and end angle of 70°, a center angle of 0°, and a transfer value that is twice the extrusion width.

**Figure 5:**
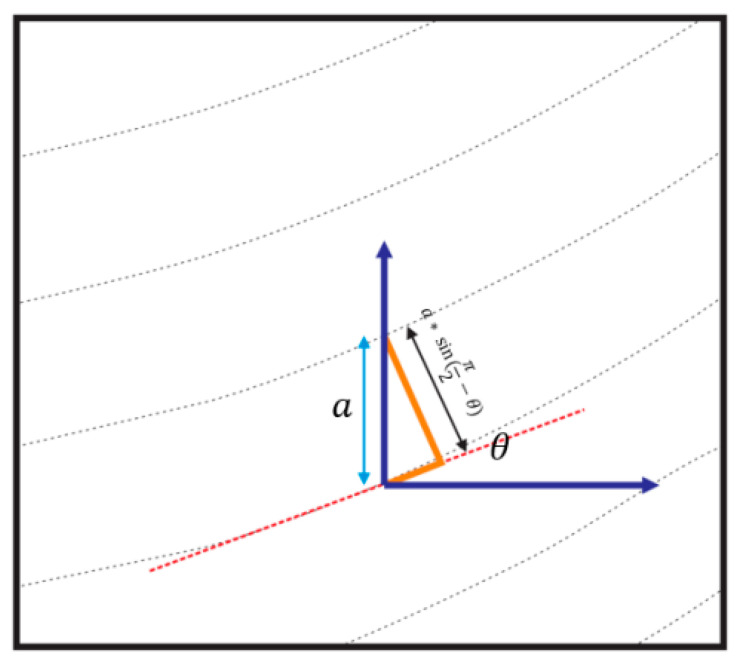
Schematic of the geometric calculation of the vertical distance between two curvilinear paths.

According to Figure 4, the volume of the polymer became uneven at different points because of the constant filament feed and the variable vertical distance between the two curves. Empty space was also observed in parts where the distance between two adjacent paths reached its maximum, indicating a lack of sufficient polymeric material. If the filament feed rose to a constant value, the polymer accumulated in areas with smaller distances and the dimensional accuracy and surface finish of the printed part decreased. To remove these defects, the filament feed was changed in proportion to the distance from the two adjacent curves. Equation (6) was used to calculate the correct filament feed.

(6) $$E=\int_{0}^{L}\left[wh/\left(\left(\pi /4\right)D^{2}\right)\right]dL$$

where w is the extrusion width, h is the layer height, D is the diameter of the filament, and dL is the differential length between two consecutive points in a curved path. The extrusion width can be replaced by the vertical distance between two adjacent curves. The geometric interpretation of the method for calculating the vertical distance between two curves at 0° is shown in Figure 5.

The formula for calculating the filament feed according to the geometric relations shown in Figure 5 is presented in Equation (7).

(7) $$E=\int_{0}^{L}\left[\left(h\right)acos\left(\theta \right)/\left(\left(\pi /4\right)D^{2}\right)\right]dL$$

where θ is the angle tangent to the curve and *a* is the transfer value of the curve. The value of *a* can be considered equal to the extrusion width.

#### 2.1.4. Computer Program for Generating G-Code

**Figure 6:**
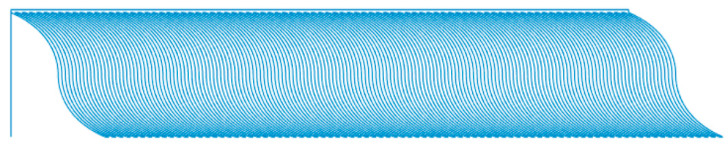
The curve path created in a layer using computer code, with a start and end angle of 70° and a middle angle of 0°.

A computer program was designed to create different curved paths. The main purpose of the program was to use different mathematical functions to move the nozzle with different parameters and generate G-code. The main features of the program include the ability to enter an optional function for the path and to change its parameters at any point. Another benefit of the program is the ability to change any of the motion parameters for the curve, such as angles and distances in each iteration of the program retrieval, through which it is possible to create complex curves. To print composite samples, the nozzle returns to its original position outside the perimeter of the part at the end of each nozzle layer due to the continuity of the fibers and the impossibility of cutting them. This situation is shown in Figure 6. The start and end commands of the program are automatically added to the main body.

## 3. Results and Discussion

The results of the created curve paths, the correction of the filament feed, and the composite samples produced are presented in this section.

### 3.1. Results of Modified G-Code for Curved Paths

**Figure 7:**
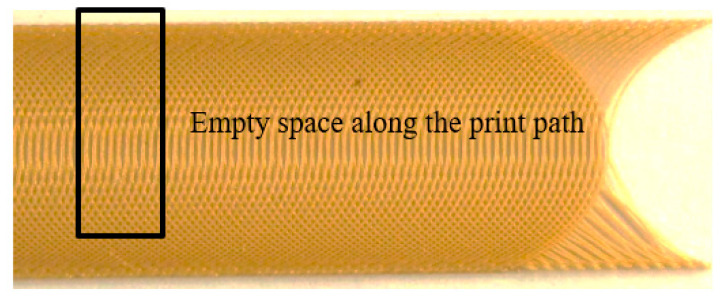
Polymeric printed sample in curved paths with a start and end angle of 70° and center angle of 0°.

The polymeric sample made by the curved paths with a start and end angle of 70° and a center angle of 0° without modifying the filament feed is shown in Figure 7.

**Figure 8:**
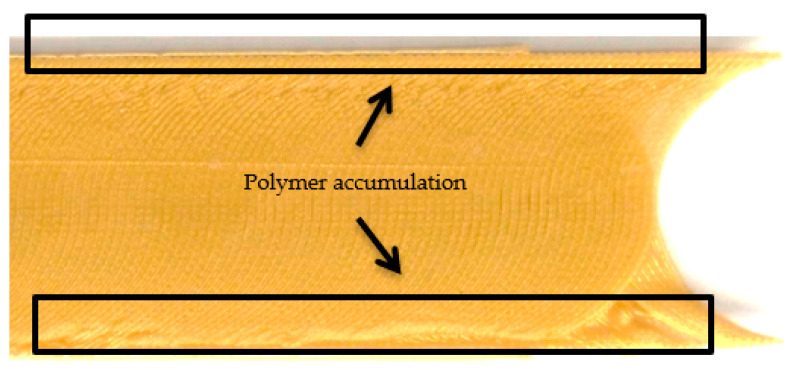
Polymer accumulation and reduced surface finish when the filament feed increased as a constant value.

The amount of polymer was constant due to the constant filament feed. When the distance between the two paths increased, there was a lack of polymer throughout the path (except for at the corners of the part) and empty spaces of different sizes were observed. If the filament feed rose to a constant value, there was no polymer deficiency at the maximum distance between the two curves, but polymer accumulation occurred at the edges of the part. As shown in Figure 8, this defect was observed at the edges of the sample due to the increase in the angle and the decrease in the vertical distance of the curve.

**Figure 9:**
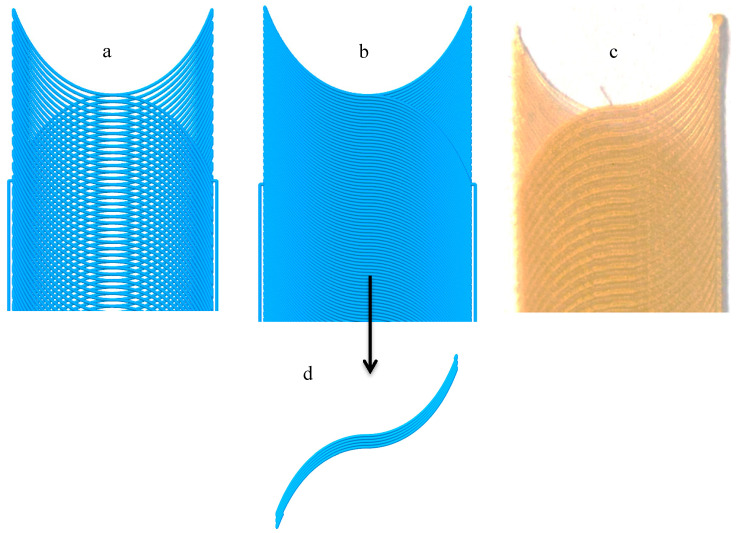
The effect of the modified filament feed on the absence of empty space and the accumulation of polymeric materials as well as the quality and surface finish of the final surface of the part. (**a**) G-code simulation for part with constant filament feed, (**b**,**d**) G-code simulation for part with variable filament feed, (**c**) Printed part with variable filament feed

The sample produced by modifying the filament feed is shown in Figure 9. As shown in Figure 7 and Figure 9a, the polymer deficiency was observed along the path when the amount of filament feed was constant. Figure 9b was obtained when the filament feed was modified according to Equation (7). As indicated in the code simulation, the thickness of the curved path varied, and polymer deficiency and accumulation were not observed anywhere. The correct printed sample is shown in Figure 9c. Proper printing of the sample, the absence of cavities, and removing polymer accumulation indicated the correctness of the filament feed.

### 3.2. Result of Printing Composites with Curved Fibers

**Figure 10:**
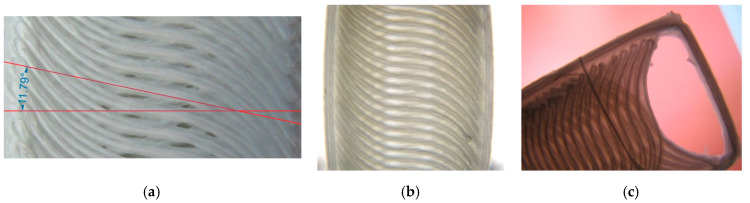
Printing composite samples with curved fibers. (**a**) The angle of the curve center at about 11${}^{{}^{\circ}}$ and (**b**,**c**) the angle of the curve center at 0${}^{{}^{\circ}}$.

Variable-stiffness composites are designed to have variable stiffness at different points, which leads to better performance than those with fixed stiffness [31]. One method to produce variable-stiffness composites is changing the angle of the fibers by considering specific curves instead of straight lines. The automatic layering technique is one method to produce parts with curved paths, but is expensive and likely to include various defects in production [31]. We examined variable-stiffness composites using the aforementioned technique. Notably, when the goal is to produce composites with continuous fibers, the filament feed must be reduced for proper printing according to the process parameters and fiber diameter. The calculations to reduce the filament feed based on the volume percentage of the fibers was studied in detail by other groups [10,11]. The printed composite sample, with a fiber angle of 0° in the middle and 70° at the corners, is shown in Figure 10.

As shown in Figure 10, by precisely controlling the filament feed, composites with curved fibers can be produced using the FDM technique. Note the deviation of the fiber angle from the specified value. In the current impregnation method, the glass fibers are pulled by the molten filament, which passes through the nozzle and feeds the outlet. As a result, a tensile force is created in the direction of the fibers that can change the radius of the curvature and crumple the fibers. To eliminate the possibility of the fibers crumpling, a margin was left around the sample to make the fibers stick better to the polymer. After observing the prototypes, we determined that the angle of the curve center, which should have been 0°, was about 11°. To fix this defect, the 0° value of the curve was replaced with a negative angle. The best curve with an angle of 0° in its center was selected after trial and error. As a result, the −5° became 0° after moving the nozzle at the specified speed.

## 4. Conclusions

The goal of this study was to properly print composites with continuous fibers in curved paths with the FDM technique. To achieve this, a novel method was designed to create curved paths and generate the necessary G-codes by modifying the filament feed. Using the proposed technique, composite samples with curved fibers were produced properly. The important conclusions from this study are presented as follows:

- By modifying the filament feed according to the distance between two adjacent paths, the empty space created by the transfer of a curve is filled and the surface smoothness of composites is improved. The filament feed can also be reduced in areas where the distance between the two curves decreases to prevent projections on the surface.
- By reducing and carefully controlling the filament feed, the continuous fibers can be correctly deposited in curved paths.
- FDM 3D printers can be used to produce variable-stiffness composites with thermoplastic materials, which has many advantages over other methods, such as low cost and higher quality.
- Due to the tension of the fibers in the current method of 3D printing, the fiber angle changes according to the nozzle speed. This defect can be reduced by modifying the program and changing the angle of the fibers in the middle of the curve.

## Figures and Tables

**Table 1:** Point coordinates, path length, and calculation of filament feed for each path.

Point	X	Y	ΔX	ΔY	Path Length (L)	Filament Feed: E (For Each Path)	Filament Feed: E (Continuous Program)
A	50	50	0	0	0	0	0
B	165	95	115	45	123.4909	5.1342	5.1342
C	70	150	–95	55	109.7725	4.5638	9.6980
D	50	50	–20	–100	101.9804	4.2399	13.9379
